# Comparative Analysis of Peptidoglycans From *Pseudomonas aeruginosa* Isolates Recovered From Chronic and Acute Infections

**DOI:** 10.3389/fmicb.2019.01868

**Published:** 2019-08-27

**Authors:** Gabriel Torrens, María Escobar-Salom, Elisabet Pol-Pol, Cristina Camps-Munar, Gabriel Cabot, Carla López-Causapé, Estrella Rojo-Molinero, Antonio Oliver, Carlos Juan

**Affiliations:** Servicio de Microbiología-Unidad de Investigación, Hospital Universitari Son Espases-Institut d’Investigació Sanitària Illes Balears (IdISBa), Palma, Spain

**Keywords:** *Pseudomonas aeruginosa*, peptidoglycan, HPLC, clinical strains, cystic fibrosis, bacteremia

## Abstract

*Pseudomonas aeruginosa* is one of the first causes of acute nosocomial and chronic infections in patients with underlying respiratory pathologies such as cystic fibrosis (CF). It has been proposed that *P. aeruginosa* accumulates mutations driving to peptidoglycan modifications throughout the development of the CF-associated infection, as a strategy to lower the immune detection hence ameliorating the chronic persistence. As well, some studies dealing with peptidoglycan modifications driving to a better survival within the host have been published in other gram-negatives. According to these facts, the gram-negative peptidoglycan could be considered as a pathogen-associated molecular pattern with very important implications regarding the host’s detection-response, worthy to dissect in detail. For this reason, in this work we characterized for the first time the peptidoglycans of three large collections [early CF, late CF and acute infection (bloodstream) *P. aeruginosa* strains] from qualitative (HPLC), quantitative and inflammatory capacity-related perspectives. The final goal was to identify composition trends potentially supporting the cited strategy of evasion/resistance to the immune system and providing information regarding the differential intrinsic adaptation depending on the type of infection. Although we found several punctual strain-specific particularities, our results indicated a high degree of inter-collection uniformity in the peptidoglycan-related features and the absence of trends amongst the strains studied here. These results suggest that the peptidoglycan of *P. aeruginosa* is a notably conserved structure in natural isolates regardless of transitory changes that some external conditions could force. Finally, the inverse correlation between the relative amount of stem pentapeptides within the murein sacculus and the resistance to immune lytic attacks against the peptidoglycan was also suggested by our results. Altogether, this work is a major step ahead to understand the biology of peptidoglycan from *P. aeruginosa* natural strains, hopefully useful in future for therapeutic alternatives design.

## Introduction

*Pseudomonas aeruginosa* is one of the main causes of opportunistic acute infection in the healthcare setting but also of chronic respiratory infection in patients with underlying chronic diseases, such as cystic fibrosis (CF) (Lyczak et al., 2002; Gellatly and Hancock, 2013; Hattemer et al., 2013). One of the most striking features of this gram-negative pathogen is its very extensive genome, which confers high capacity for adaptation to stressful conditions, hence entailing an outstanding capacity for antibiotic resistance development. This worrying feature, achieved through chromosomal mutations and/or horizontal acquisition of resistance determinants such as β–lactamases, makes of *P. aeruginosa* one of the most difficult microorganisms to defeat through classical antibiotic treatments. In fact, *P. aeruginosa* is becoming virtually untreatable in a growing number of cases nowadays (Poole, 2011; Oliver et al., 2015; López-Causapé et al., 2018; Mulani et al., 2019). Additionally, as stated before, this adaptation capacity allows *P. aeruginosa* to grow in very hostile environments, an example of which could be the lungs of CF patients, in which bacteria must cope with many aggressive conditions. Among these, we could cite the limitation of certain nutrients and oxygen, the continuous influx of immune system compounds, the extreme osmolarity and the antibiotic treatments routinely administered to the patients (Yang et al., 2011; Gellatly and Hancock, 2013; Moradali et al., 2017). To survive in this situation, *P. aeruginosa* has been described to progressively accumulate different chromosomal mutations that allow a better adaptation to such a particular environment. Obviously, the mutations driving to antimicrobial resistance stand out among them, but a wide array of additional features have been reported to be positively selected during the chronic process. For instance, the increase in the mutation frequency, the loss of virulence factors, appearance of auxotrophic and small colony variants, alginate hyper-production driving to increased capacity for biofilm formation, loss of flagellar motility, etc. (Luzar et al., 1985; Mahenthiralingam et al., 1994; Oliver et al., 2000; Feliziani et al., 2010; López-Causapé et al., 2017; Faure et al., 2018). Among these mutation-driven features, the reduction in the immuno-stimulatory capacity (closely related to virulence and inflammation) has been proposed to be very important, since a decreased detection by the immune system seems to ameliorate chronic persistence. In this regard, some works have shown that lipopolysaccharide and peptidoglycan variants with reduced inflammatory capacity can be selected in CF strains (Cigana et al., 2009, 2011). Nevertheless, although it is unanimously accepted that the virulence attenuation is a hallmark of *P. aeruginosa* chronic strains (Cullen and McClean, 2015; Faure et al., 2018), the reduction in the inflammatory capacity is far from being a uniform feature of them. In fact, several works display contradictory results in this regard (Cigana et al., 2009; Hawdon et al., 2010; Di Lorenzo et al., 2015; LaFayette et al., 2015; Torrens et al., 2019). As well, the active search for peptidoglycans with reduced or increased inflammatory capacity has never been conducted in large numbers of *P. aeruginosa* clinical isolates, but only in punctual strains (Cigana et al., 2009). In fact, the descriptive studies on the characteristics of peptidoglycans purified from wide collections of other gram-negatives are also very scarce (Boltaña et al., 2011; Wu et al., 2015).

In spite of its starring role as target for β–lactams, the gram-negative peptidoglycan has been poorly studied from other perspectives, for instance as a pathogen-associated molecular pattern (PAMP) with important implications in issues as the mentioned immune activation (Cava and de Pedro, 2014; van Teeseling et al., 2017; Irazoki et al., 2019). Thus, it has not been widely studied in terms of interplay with virulence, inflammatory capacity or adaptation to the environment, at least in gram-negatives, because of the obvious protection exerted by the outer membrane and lipopolysaccharide (Davis and Weiser, 2011; Cava and de Pedro, 2014; Zhao et al., 2017; Juan et al., 2018). Besides, although not as numerous as regarding gram-positives, in gram-negatives there are some evidences of peptidoglycan modifications helping the pathogen for infection establishment and development in punctual mutants (Rosenthal et al., 1982; Swim et al., 1983; Costa et al., 1999; Dillard and Hackett, 2005; Chaput et al., 2006; Humann and Lenz, 2009; Wang et al., 2009, 2010, 2012; Davis and Weiser, 2011; Sukhithasri et al., 2013; Hernández et al., 2015; Schaub and Dillard, 2019a, b). Thus, although some elements in this essential structure have been identified as potential anti-virulence targets in different gram-negative species (Juan et al., 2018), the works characterizing the peptidoglycan in large collections of *P. aeruginosa* clinical strains are non-existent.

For all these antecedents and reasons, we wanted to characterize the peptidoglycans purified from extensive collections of *P. aeruginosa* clinical strains (CF vs. bacteremia) from the quantitative and qualitative perspectives, including the determination of their pro-inflammatory capacity. The goal was to identify peptidoglycan composition trends potentially supporting the cited strategies of evasion/resistance to the immune system and providing information regarding the *P. aeruginosa* differential adaptation depending on the type of infection. Our analysis was focused on the intrinsic phenotypes that the mentioned clinical strains constitutively display, regardless of transitory changes that their peptidoglycans could show in response to certain external factors. All these issues have never been studied before in large numbers of clinical strains, making of this work a pioneer study in the field.

## Materials and Methods

### Bacterial Strains

Besides the reference strains (*P. aeruginosa* PAO1 and PA14 and *Escherichia coli* XL-1 blue), the previously described mutant of *P. aeruginosa* PAOΔdacBΔdacCΔpbpG (Ropy et al., 2015) was used for comparative purposes and as a control for our procedures’ capacity to detect differences in the muropeptides composition. The clinical strains used in this work belong to two previously described collections of clinical isolates, recently analyzed in another work of our group and displayed in the Supplementary Data Set S1 (Torrens et al., 2019). First, twelve strains isolated from bacteremia patients and belonging to a Spanish multicenter study (Cabot et al., 2011) were included. Second, we used twelve pairs of CF sequential isogenic isolates [early-late isolate, obtained with a difference of at least 3 years from a total of 10 patients (thus, from two of the patients, two pairs of isolates were obtained on each, at different time points)], proceeding from a previously published work (López-Causapé et al., 2013). The previously determined values of ceftazidime Minimum Inhibitory Concentration (MIC, mg/L), and the percentages of bacterial survival after treatments with: (i) lysozyme (25 mg/L) + colistin (0.025 mg/L) (ii) Peptidoglycan Recognition Protein 1, PGLYRP1 (50 mg/L) + colistin (0.025 mg/L) and (iii) PGLYRP2 (50 mg/L) + colistin (0.025 mg/L) are also displayed in the Supplementary Data Set S1 (Cabot et al., 2011; López-Causapé et al., 2013; Torrens et al., 2019).

### Cell Culture and Analysis of Inflammatory Response Caused by Peptidoglycans

The A549 human type II alveolar epithelial cell line was purchased from Cell Line Service (Germany) and used between the passages 3 and 30. The cells were maintained in RPMI-1640 (Biowest) supplemented with 10% of heat-inactivated fetal bovine serum, 10mM HEPES, 2 mM L-glutamine and 1X antibiotic-antimycotic solution (Biowest). Cells were seeded at approximately 1 × 10^5^ cells per well in 24-wells plates. The day after, they were stimulated during 20 h with 20 μg/mL of the purified mutanolysin-digested peptidoglycans (see next paragraph) dissolved in the mentioned medium without fetal bovine serum. To assess the elicited inflammatory response, the secretion of interleukin IL-8 was used as indicator (Hawdon et al., 2010; Pérez-Gallego et al., 2016; Torrens et al., 2017) through the Human IL-8/NAP-1 Instant ELISA kit (eBioscience-Affymetrix), following the manufacturer’s instructions. The supernatants of cells after stimulation with peptidoglycans were used as samples and those from wells with medium without any stimuli were used as basal controls. The supernatants of wells stimulated with 20 μg/mL of muramyl dipeptide (MDP) or its synthetic derivative L-18 MDP (Invivogen) were used as positive controls (Matsumoto et al., 1981; Girardin et al., 2003a). The assays were repeated at least in three wells from each of three independent 24-wells plates for each peptidoglycan. To discard a cytotoxic effect of the incubation with the purified peptidoglycans over the cells, the Cytotoxicity Detection Kit PLUS (Roche) was used to quantify the released Lactate DesHydrogenase (LDH), following the manufacturer’s instructions.

### Peptidoglycans Purification for Cell Culture Stimulation

The peptidoglycans from the reference and clinical strains were extracted following previously described protocols with slight modifications (Zhang et al., 2013; Ropy et al., 2015). The different strains were grown overnight in 1L of LB broth at 37°C and 180 rpm. The cells were suspended in double-distilled water. An equal volume of boiling 20% SDS solution was slowly added, and the final suspension was kept boiling for 12 h with stirring. The suspensions were centrifuged at 18000 *g* for 45 min to collect the sacculi fraction, which was then washed with warm sterile double-distilled water at least three times. Pellets were suspended in 10 mM Tris-HCl (pH 7.6) supplemented with 0.5 mM CaCl_2_ and 2.5 mM MgCl_2_, and treated with α-amylase (Sigma-Aldrich), Turbo DNAse (Ambion), RNAse (Sigma-Aldrich) for 2 h at 37°C, and finally with pronase E (Merck) at 60°C for 90 min. The enzymes were inactivated by adding an equal volume of 20% SDS solution and boiling for 10’, and next, peptidoglycans were collected and washed as described above. After that, peptidoglycans were lyophilized for weighing and quantification. Samples were treated with 8 M LiCl for 1 h at room temperature. The peptidoglycans were centrifuged and washed three times, and treated with 100 mM EDTA for 1 h at room temperature. Samples were centrifuged and washed as above, and treated with acetone for 1 h at room temperature. After at least three washes, the pellets were suspended in 50 mM NaH_2_PO_4_ (pH 4.9) and digested with mutanolysin (Sigma-Aldrich) overnight. Next, the enzyme was inactivated and the samples were centrifuged for 5 min to remove insoluble debris. Finally, the supernatants were 0.22 μm-filtered. The *E*-toxate reagent (Sigma-Aldrich) was used to check the absence of endotoxin contamination, following the manufacturer’s instructions.

### Preparation of Peptidoglycans for HPLC Analysis

The peptidoglycan purification protocol was similar to that described above, with some exceptions: unless indicated, the overnight cultures were diluted 1:100 and incubated (37°C, 180 rpm) to reach the exponential growth phase (OD_600_ nm up to 0.75), in a final volume of 250 mL. The RNAse, DNAse, LiCl, EDTA and acetone treatments were omitted. After the resuspension in 50 mM phosphate buffer (pH 4.9), samples were digested with 100 μg/ml mutanolysin (Sigma-Aldrich) at 37°C overnight. The enzyme was then inactivated by 10 min boiling, and centrifuged at 18000 *g* to remove insoluble debris. The supernatant was mixed with 1/3 volume of 0.5 M sodium borate buffer (pH 9.0) and all the sample was reduced with excess of sodium borohydride (NaBH_4_) for 30 min at room temperature. The pH was adjusted to 3 with orthophosphoric acid, obtaining a final volume of 500–510 μL. All samples were 0.22-μm filtered and injected (100 μL) into the HPLC device. Separations were performed on a Breeze 2 HPLC system, consisting of a 1525 binary HPLC pump model code 5CH (Waters), a UV-visible detector 2489 (Waters), a manual injector model 7725i (Rheodyne), and an Aeris Peptide XB-C18, 3.6 μm, 250 by 4.6 mm reverse-phase column (Phenomenex). Separation of individual components (muropeptides) of peptidoglycan was performed in a linear gradient, the column was equilibrated at 45°C, and the eluted compounds were detected at a wavelength of 204 nm (detection limit: any peak higher than 0.05 AU was used for quantification). The mobile-phase (A = 50 mM sodium phosphate [pH 4.35]; B = 75 mM sodium phosphate, 15% methanol [pH 4.95]) gradient consisted of elution at 1.0 ml/min with 100% A for 5 min, followed by a 60-min linear gradient to 0% A/100% B and then 100% B for 5 min. The identification of individual muropeptides was carried out according to retention time, using a comparison analysis with the retention times of known muropeptides. The complete list of known muropeptides used for quantification, together with representative chromatograms is shown in the Supplementary Figure S1. When a difference was found in the retention time of a particular peak, this peak was purified, and the structure was confirmed or characterized by matrix-assisted laser desorption ionization–time of flight (MALDI-TOF) mass spectrometry with the autoflex spectrometer (Bruker Daltonics). The relative abundances of muropeptides present in each sample were determined by integrating their respective areas of absorption (Breeze 2, Waters program) applying the correction factors described by Glauner for UV detection (Glauner, 1988), and grouped and expressed as the molar fraction (mol%) relative to the total content (Glauner, 1988). The average number of disaccharide units/chain was calculated by dividing the mol% of anhydro-muropeptide (proportional to chain ends) by the total molar amount of muropeptides in the digested peptidoglycan. The degree of cross-linking was obtained by calculating the mol% of dimers and trimers with respect to total muropeptides, as previously described by Glauner (1988) and Glauner et al. (1988). Each peptidoglycan extraction and HPLC were performed at least in independent duplicates.

### Peptidoglycan Quantification

The peptidoglycans from the studied strains were quantified by means of the titration of meso-diaminopimelic acid (mDAP) concentration, following previously described protocols (Dörr et al., 2014; Torrens et al., 2017). Briefly, the murein sacculi from 250 mL LB broth cultures in late exponential phase (adjusted to an OD_600_ = 0.8 to normalize the number of cells) were hydrolyzed for 18 h with HCl 6M at 100°C. Afterward, the samples were liophilyzed, resuspended in water and treated with ninhydrin reagent (250 mg of ninhydrin disolved in 4 mL of 0.6 M phosphoric acid +6 ml of acetic acid glacial) for 5 min at 100°C. OD_436_ was measured and concentration of muropeptides was calculated using a mDAP standard curve. Each peptidoglycan quantification was performed in three independent replicates, and finally expressed as the mean amount of mDAP per cell.

### Data Analysis

The GraphPad Prism 5 software was used for graphical representation and statistical analysis. Quantitative variables were compared using Student’s *t*-test or Mann–Whitney *U* test as appropriate. Once checked the Gaussian distribution (Shapiro–Wilk Normality test) of the values, the quantitative variables were compared using the unpaired two-tailed Student’s *t*-test (except in the comparison of the early with late CF isolates, in which a paired test was applied). When multiple tests had to be done with the same data set with regards to reference strain, the One-way ANOVA with *post hoc* Tukey’s multiple comparison test was performed. For all these analysis, a *P*-value of <0.05 was considered statistically significant.

To assess the potential correlation between HPLC-derived parameters and phenotypes of susceptibility/resistance to aggressions against peptidoglycan [ceftazidime MICs and percentages of bacterial survival after treatments with lysozyme/PGLYRP1/PGLYRP2 + colistin (Torrens et al., 2019)], the bivariate associations were evaluated by Spearman’s correlation coefficients. The indicated variables were dichotomized using the median and the comparisons between groups were analyzed using the Mann–Whitney *U* test. A two-tailed *p*-value <0.05 was considered statistically significant. For these statistical analyses, the SPSS software version 23.0 was used.

## Results

### The HPLC Analysis of Peptidoglycans From Bacteremia vs. Cystic Fibrosis Strains Reveals a High Inter-Collection Uniformity, With Punctual Strain-Specific Particularities

The composition of the peptidoglycans from reference (PAO1 and PA14) and the whole set of clinical strains was studied through muropeptide HPLC analysis, and the results are shown in the Table 1 (bacteremia) and Table 2 (CF). Representative chromatograms from different strains are shown in the Supplementary Figure S1, together with the whole array of muropeptides that was considered for the quantification of the different parameters displayed in these Tables. Some of the parameters showed very little differences when comparing the strains within a collection, but also when trying to establish patterns among collections. For instance, the relative abundance of monomers, dimers, trimers and the cross-linking levels were very uniform. In fact, when statistically analyzing these parameters from the three collections (bloodstream, early CF and late CF, Table 3), no significant differences were found (*P-*value > 0.05 in all the cases). Moreover, although the rest of parameters were more variable, the differences among collections were neither statistically significant for any of them (*P*-value > 0.05, Table 3). Hence, no trends could be drawn based on the different origin of our stains (acute vs. early CF vs. late CF). However, some interesting isolate-specific features were found, as can be observed in the Tables 1–3. For instance, (i) a higher proportion of muropeptides bound to OprI (ortholog of Braun’s Lipoprotein, Lin et al., 2010), i.e., circa twofold with regards to the mean value of the three collections, and (ii) a lower proportion of *meso*-diaminopimelic acid-*meso*-diaminopimelic acid (mDAP-mDAP) bridges (circa 10-fold less with regards to the mean value of the three collections), in the bacteremia strains PABA-48 and PABA-211. Besides, the bacteremia strain PABA-19 showed a higher proportion of pentapeptide stems in the murein sacculus (circa threefold with regards to the mean value of the three collections, Table 3). In the case of the CF strains, the proportion of mDAP-mDAP bridges was also circa 10-fold lower compared to the mean values of the three collections for several of them (PAFQ06-03, PAFQ10-03, PAFQ16-03, PAFQ16-10, PAFQ21-1088-10, and PAFQ24-04). On the contrary, the strain PAFQ21-1088-03 showed a circa fivefold increase in this same parameter. Interestingly, this strain also stood out because its crosslinking degree and proportion of stem pentapeptides were the two highest values among all the strains. Finally, the strains PAFQ11-10, PAFQ21-1109-10 and PAFQ24-10 also showed an increased value for this last parameter (reaching circa 2–3–fold compared to the mean value of the three collections, Table 3). Dealing with the rest of parameters, the sugar chains mean length was more constant in the bacteremia (range 13.6–28.3 disaccharide units) than in the CF strains (11.8–33.5), but without a trend when comparing early/late isolates. Finally, as can be observed in the Tables 1–3, the mean values of all the parameters from the three collections were very similar to those of the reference strains PA14 and PAO1, which were virtually identical for the majority of the data, with the only exception of an increased relative abundance of stem pentapeptides in this latter strain (circa twofold).

**TABLE 1:** HPLC analysis of muropeptides prepared from the peptidoglycans of the reference and bacteremia strains studied in this work.

**Strain**	**Relative abundance (mol%) of muropeptide^a^**							**Cross-link^b^ (%)**	**Peptidoglycan length^c^**
	
	**Mono**	**Di**	**Tri**	**D-D**	**Lpp**	**Anh**	**Penta**		
PAO1	65.46	32.51	2.04	1.55	4.86	4.25	2.83	36.58	23.54
PA14	66.08	32.37	1.56	1.73	3.75	4.25	1.50	35.48	24.11
PAOΔdacB ΔdacCΔpbpG	55.40	39.71	4.89	0.17	0.34	5.59	79.83	49.49	17.90
PABA-12	65.71	32.06	2.22	1.44	3.47	5.07	3.02	36.51	20.14
PABA-19	70.15	27.87	1.98	1.31	5.66	7.67^∗^	6.98^∗^	31.82	13.16^∗^
PABA-28	64.03	33.72	2.25	0.65	5.90	5.92	0.10^∗^	38.23	17.18
PABA-37	64.16	33.56	2.27	0.73	3.76	4.05	0.70	38.11	24.71
PABA-43	62.61	34.47	2.92	1.10	4.97	5.93	1.08	40.31	16.88
PABA-48	67.95	31.44	0.65^∗^	0.05^∗^	11.84^∗^	4.39	0.63	32.65	28.27
PABA-63	69.14	29.39	1.47	3.34	2.50	6.81	4.77	32.33	15.02
PABA-100	63.63	34.09	2.28	1.14	5.81	4.69	0.10^∗^	38.65	21.72
PABA-120	65.78	32.25	1.97	1.08	4.49	3.90	1.48	36.19	25.63
PABA-142	64.16	33.44	2.40	0.85	5.21	4.58	0.10^∗^	38.24	21.84
PABA-181	65.82	31.92	2.26	0.42	1.93	4.73	0.56	36.44	22.35
PABA-211	70.40	28.36	1.24	0.10^∗^	12.08^∗^	5.84	2.19	30.84	17.20

*^*a*^Mol%, molar fraction; Mono, monomers; Di, dimers; Tri, trimers; D-D, muropeptides having *meso*-diaminopimelic acid - *meso*-diaminopimelic acid (mDAP–mDAP) crosslinking; Lpp, muropeptides bound to C-terminal Arg-Lys dipeptide of OprI lipoprotein; Anh, muropeptides having anhydro-1,6-anhydromuramic acid; Penta, muropeptides having a pentapeptide stem (all these pentapeptides were shown to possess glycine instead of D-alanine in terminal position). ^*b*^Cross-link, degree of peptidoglycan cross-linking (percentage). ^*c*^Average number of disaccharide (*N*-acetylglucosamine and *N*-acetylmuramic acid) units in the sacculus sugar chains of each strain. The displayed value of each parameter and strain is the mean obtained from two independent replicates of peptidoglycan extraction and HPLC analysis. In all the cases, the standard deviation (SD) was below 20% of each value (data not shown). ^∗^Statistically significant (One way ANOVA with Tukey’s *post Hoc* test, *P*-value < 0.05), compared to PAO1. The previously described mutant of *P. aeruginosa* PAOΔdacBΔdacCΔpbpG (Ropy et al., 2015) was used for comparative purposes and as a control for our procedures’ capacity to detect differences in the muropeptides composition.*

**TABLE 2:** HPLC analysis of muropeptides prepared from the peptidoglycans of the cystic fibrosis strains studied in this work.

**Strain**	**Relative abundance (mol%) of muropeptide^a^**							**Cross-link^b^ (%)**	**Peptidoglycan length^c^**
	
	**Mono**	**Di**	**Tri**	**D-D**	**Lpp**	**Anh**	**Penta**		
PAO1	65.46	32.51	2.04	1.55	4.86	4.25	2.83	36.58	23.54
PA14	66.08	32.37	1.56	1.73	3.75	4.25	1.50	35.48	24.11
PAOΔdacB ΔdacCΔpbpG	55.40	39.71	4.89	0.17	0.34	5.59	79.83	49.49	17.90
PAFQ05-03	59.45	37.11	3.44	0.49	4.98	6.99	1.00	43.99	14.40
PAFQ05-11	67.56	29.43	3.02	0.41	3.72	5.47	0.50^∗^	35.46	18.39
PAFQ06-03	66.79	30.96	2.25	0.22^∗^	8.20^∗^	5.12	3.47	35.46	19.64
PAFQ06-10	66.49	31.45	2.06	0.81	7.56	3.79	2.02	35.57	26.58
PAFQ10-03	66.52	31.80	1.68	0.10^∗^	3.81	2.99	0.57	35.16	33.52
PAFQ10-11	67.37	31.39	1.24	0.63	5.81	4.02	3.58	33.88	28.05
PAFQ11-03	61.94	34.95	3.12	1.47	3.62	7.45^∗^	1.49	41.18	13.82^∗^
PAFQ11-10	69.22	29.36	1.41	1.26	4.42	3.52	6.28^∗^	32.19	28.72
PAFQ12-146-07	63.24	34.77	1.99	1.39	4.67	3.35	0.69	38.75	30.54
PAFQ12-146-10	64.04	34.94	1.02	1.31	7.29	4.68	2.16	36.98	23.47
PAFQ12-299-03	59.05	37.73	3.22	0.54	5.97	6.42	2.98	44.17	15.60
PAFQ12-299-06	57.72	38.58	3.70	1.21	4.05	6.36	2.93	45.99	15.77
PAFQ15-03	66.25	31.65	2.10	0.46	3.27	7.05	2.78	35.84	14.57
PAFQ15-10	62.16	35.66	2.18	3.18	3.82	5.61	2.36	40.02	18.19
PAFQ16-03	62.78	34.66	2.55	0.20^∗^	6.71	6.28	1.53	39.77	16.86
PAFQ16-10	60.11	36.74	3.15	0.10^∗^	4.36	5.98	1.79	43.04	16.85
PAFQ21-1088-03	55.72^∗^	38.02	6.26^∗^	4.89^∗^	5.16	7.58	7.10^∗^	50.54^∗^	17.97
PAFQ21-1088-10	64.95	32.26	2.79	0.21^∗^	3.61	7.31	1.92	37.84	14.06
PAFQ21-1109-05	65.22	32.31	2.47	0.70	4.57	5.91	1.76	37.25	17.06
PAFQ21-1109-10	65.14	32.89	1.98	0.43	5.67	6.13	4.87	36.84	16.37
PAFQ24-04	69.66	29.04	1.30	0.10^∗^	4.95	5.05	2.02	31.65	25.40
PAFQ24-10	68.37	29.76	1.87	1.63	5.19	9.41^∗^	5.28	33.50	11.75^∗^
PAFQ28-06	67.63	30.59	1.79	0.76	3.97	6.23	2.77	34.16	16.71
PAFQ28-10	66.59	31.57	1.84	0.77	4.41	5.45	2.37	35.25	19.79

*^*a*^Mol%, molar fraction; Mono, monomers; Di, dimers; Tri, trimers; D-D, muropeptides having mDAP–mDAP crosslinking; Lpp, muropeptides bound to C-terminal Arg-Lys dipeptide of OprI lipoprotein; Anh, muropeptides having anhydro-1,6-anhydromuramic acid; Penta, muropeptides having a pentapeptide stem (all these pentapeptides were shown to possess glycine instead of D-alanine in terminal position). ^*b*^Cross-link, degree of peptidoglycan cross-linking (percentage). ^*c*^Average number of disaccharide (*N*-acetylglucosamine and *N*-acetylmuramic acid) units in the sacculus sugar chains of each strain. The displayed value of each parameter and strain is the mean obtained from two independent replicates of peptidoglycan extraction and HPLC analysis. In all the cases, the SD was below 20% of each value (data not shown). ^∗^Statistically significant (One way ANOVA with Tukey’s *post Hoc* test, *P*-value < 0.05), compared to PAO1. The previously described mutant of *P. aeruginosa* PAOΔdacBΔdacCΔpbpG (Ropy et al., 2015) was used for comparative purposes and as a control for our procedures’ capacity to detect differences in the muropeptides composition.*

**TABLE 3:** Values of the parameters obtained from the HPLC-analyzed peptidoglycans purified from the collections studied in this work.

**Collection**	**Relative abundance (mol%) of muropeptide^a^**							**Cross-link^b^ (%)**	**Peptidoglycan length^c^**
	
	**Mono**	**Di**	**Tri**	**D-D**	**Lpp**	**Anh**	**Penta**		
PAO1	65.46	32.51	2.04	1.55	4.86	4.25	2.83	36.58	23.54
PA14	66.08	32.37	1.56	1.73	3.75	4.25	1.50	35.48	24.11
Bacteremia	66.1 ± 2.6 (65.7)^*d*^	31.9 ± 2.2 (32.1)	2.0 ± 0.61 (2.2)	1.0 ± 0.86 (1.0)	5.6 ± 3.2 (5.1)	5.3 ± 1.1 (4.9)	1.8 ± 2.1 (0.9)	35.9 ± 3.1 (36.5)	20.3 ± 4.6 (20.9)
Early CF	63.7 ± 4.1 (64.2)	33.6 ± 3.0 (33.5)	2.7 ± 1.3 (2.3)	0.95 ± 1.3 (0.5)	4.9 ± 1.4 (4.8)	6.0 ± 1.6 (6.3)	2.3 ± 1.7 (1.9)	39.0 ± 5.3 (38.0)	19.6 ± 6.5 (16.9)
Late CF	64.9 ± 3.5 (65.8)	32.8 ± 3.0 (31.9)	2.2 ± 0.82 (2.0)	1.0 ± 0.83 (0.8)	5.0 ± 1.34 (4.4)	5.6 ± 1.6 (5.5)	3.0 ± 1.7 (2.4)	37.2 ± 4.0 (36.2)	19.8 ± 5.6 (18.3)
Collections’ mean ± SD	64.9 ± 1.2	32.7 ± 0.85	2.3 ± 0.36	0.99 ± 0.02	5.1 ± 0.37	5.6 ± 0.35	2.3 ± 0.60	37.3 ± 1.55	19.9 ± 0.36

*^*a*^Mol%, molar fraction; Mono, monomers; Di, dimers; Tri, trimers; D-D, muropeptides having mDAP–mDAP crosslinking; Lpp, muropeptides bound to C-terminal Arg-Lys dipeptide of OprI lipoprotein; Anh, muropeptides having anhydro-1,6-anhydromuramic acid; Penta, muropeptides having a pentapeptide stem. ^*b*^Cross-link, degree of peptidoglycan cross-linking (percentage). ^*c*^Average number of disaccharide (*N*-acetylglucosamine and *N*-acetylmuramic acid) units in the sacculus sugar chains of each strain. ^*d*^The values in the table are the means ± SDs obtained from each parameter and collection, whereas the numbers within parenthesis correspond to the medians. The last row correspond to the mean values ± SDs calculated from all the parameters and collections.*

In spite of these strain-specific particularities, the HPLC-derived parameters were surprisingly uniform among all the collections (Table 3). This was a perhaps unexpected circumstance given the presumable high level of adaptation of each group of strains to their respective profiles of infection (acute vs. early CF vs. late CF). For this reason we wanted to ascertain if the *P. aeruginosa* peptidoglycan could also display an increased stability even in different metabolic stadiums, represented by the growth phases (exponential vs. stationary). Thus, we proceeded to purify the murein sacculus from PAO1 cultures grown at exponential phase (OD_600_:0.75) vs. those grown overnight (OD_600_ > 3), and the same for the *E. coli* XL1 strain, to finally analyze the derived muropeptides through HPLC. As can be observed in the Table 4, the relative abundance of monomers, dimers and trimers was quite stable when comparing the two growth phases of PAO1, whereas a significant reduction for the sugar chains mean length was observed for the stationary phase (19.81 vs. 12.33, *P* < 0.05). On the contrary, the proportion of mDAP-mDAP bridges, muropeptides bound to OprI and stem pentapeptides were increased in the stationary phase in PAO1. The crosslinking level was also slightly increased in the stationary phase (36.81 vs. 37.92, *P* > 0.05) in PAO1. Our results for XL1 blue evidenced more exaggerated differences between the two phases regarding the relative abundances of monomers, dimers, trimers, mDAP-mDAP bridges and cross-linking levels, but a more stable proportion of muropeptides bound to Braun’s lipoprotein (Table 4). Surprisingly, in contrast with previous publications (Pisabarro et al., 1985), the sugar chains length in our *E. coli* strain was oddly stable between the two phases.

**TABLE 4:** HPLC analysis of muropeptides prepared from the peptidoglycans of *P. aeruginosa* PAO1 reference strain at exponential vs. stationary growth phases.

**Strain and growth phase**	**Relative abundance (mol%) of muropeptide^a^**							**Cross-link^b^ (%)**	**Peptidoglycan length^c^**
	
	**Mono**	**Di**	**Tri**	**D-D**	**Lpp**	**Anh**	**Penta**		
PAO1 Exponential (OD_600_:0.75)	65.20	32.77	2.02	1.77	4.83	5.18	1.99	36.81	19.81
PAO1 Stationary (OD_600_ > 3)	64.15	33.78	2.10	2.65	12.35^∗^	8.17^∗^	8.38^∗^	37.92	12.33^∗^
XL1 blue Exponential (OD_600_:0.75)	63.25	35.30	1.47	4.23	6.95	5.11	7.10	38.25	19.60
XL1 blue Stationary (OD_600_ > 3)	59.76^∗^	36.57	3.67^∗^	12.2^∗^	8.30	5.22	5.33	43.90^∗^	19.20

*^*a*^Mol%, molar fraction; Mono, monomers; Di, dimers; Tri, trimers; D-D, muropeptides having mDAP-mDAP crosslinking; Lpp, muropeptides bound to C-terminal Arg-Lys dipeptide of OprI or Braun’s lipoprotein; Anh, muropeptides having anhydro-1,6-anhydromuramic acid; Penta, muropeptides having a pentapeptide stem (all these pentapeptides were shown to possess glycine instead of D-alanine in terminal position). ^*b*^Cross-link, degree of peptidoglycan cross-linking (percentage). ^*c*^Average number of disaccharide (*N*-acetylglucosamine and *N*-acetylmuramic acid) units in the sacculus sugar chains of each strain. The displayed value of each parameter and strain is the mean obtained from two independent replicates of peptidoglycan extraction and HPLC analysis. In all the cases, the SD was below 20% of each value (data not shown). ^∗^Statistically significant, *P*-value < 0.05 in the unpaired Student *t*-test, when comparing a given parameter between the two growth phases separately for each species.*

### Correlation Between HPLC-Derived Parameters and Susceptibility to Aggressions Against Peptidoglycan

**FIGURE 1:**
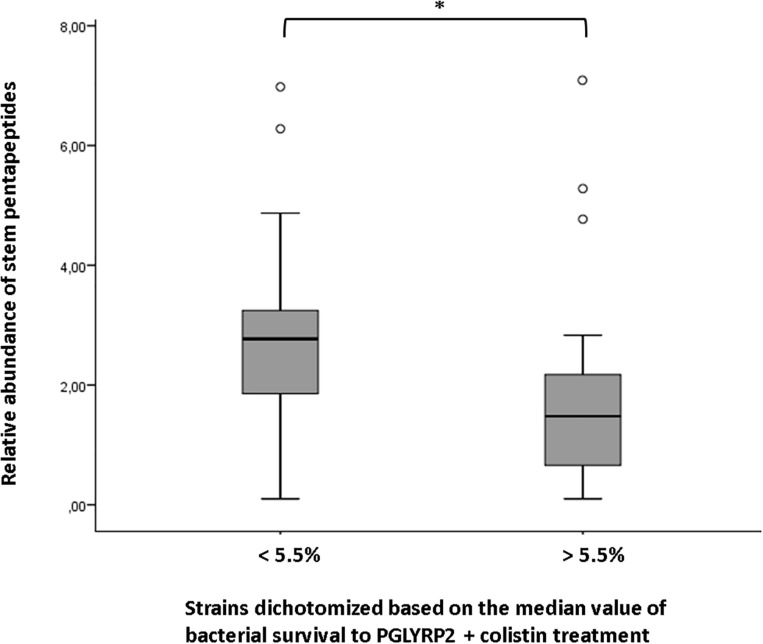
Correlation between the relative abundance of stem pentapeptides within murein sacculus and survival to PGLYRP2 (50 mg/L) + colistin (0.025 mg/L) treatment. The whole set of strains studied in this work was dichotomized based on the median value of survival to the mentioned treatment (data obtained from Torrens et al., 2019, Supplementary Data Set S1). The horizontal bar within the boxes represents the mean value of relative abundance of stem pentapeptides, whereas the upper and lower sides of the boxes correspond to the interquartile (Q1–Q3) range. The bars outside the boxes represent the maximum and minimum value of each group of strains, whereas the white dots represent the outliers. ^∗^*P* < 0.05 in the Mann–Whitney *U* test.

Although not enough to draw any trend among collections, the existence of an appreciable degree of intra-collections variability in the HPLC-derived parameters, made us think about the possibility of linking certain of the structural/compositional features with previously published phenotypes from the same strains (Cabot et al., 2011; López-Causapé et al., 2013; Torrens et al., 2019). In this regard, we sought to see if the phenotypes of susceptibility to different types of aggressions against the peptidoglycan, i.e., ceftazidime MIC and bacterial survival to the treatments of lysozyme + colistin, PGLYRP1 + colistin or PGLYRP2 + colistin (Torrens et al., 2019) (Supplementary Data Set S1), could be correlated with any of the HPLC-derived parameters. The search for correlations by means of the Spearman’s coefficients only provided a statistically significant inverse correlation between the survival to PGLYRP2 + colistin treatment and the relative abundance of stem pentapeptides (Spearman rank correlation coefficient −0.394; *p*-value 0.014). Although not significant, the inverse correlation between this last parameter and the survival to lysozyme + colistin treatment was not far from significance (Spearman rank correlation coefficient −0.29; *p*-value 0.078). Moreover, as can be observed in the Figure 1, in which the whole set of strains was dichotomized based on the median value of survival to PGLYRP2 + colistin treatment (strains below 5.5% of survival vs. strains above 5.5%), the difference between the mean values of relative abundance of stem pentapeptides from the two groups was statistically significant (*p*-value 0.038). Thus, our results suggest that an inverse correlation between the relative amount of stem pentapeptides within the murein sacculus and the resistance to PGLYRP2 once this protein reaches the peptidoglycan, does exist.

### Quantification of the Amount of Peptidoglycan per Cell in the *P. aeruginosa* Strains From Bacteremia vs. Cystic Fibrosis Reveals Minor Differences Among Collections

**FIGURE 2:**
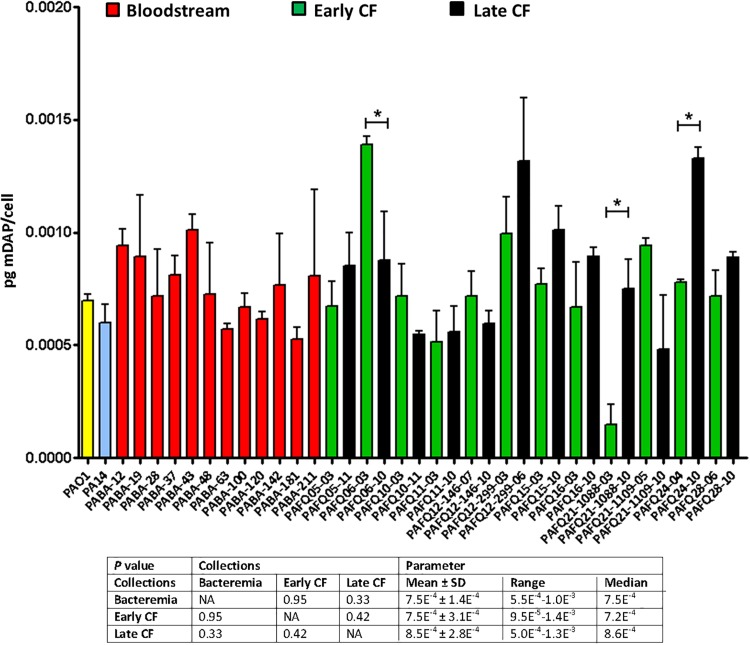
Peptidoglycan quantification through the titration of meso-diaminopimelic acid (mDAP, pg/bacterial cell) in the strains studied in this work. Each column represents the mean value of three independent determinations for each specific strain, whereas the error bar represents the standard deviation (SD). In the CF pairs of isogenic isolates, the green columns correspond to early and the black to late isolates, respectively. All the bacteremia strains are displayed with the red columns. The asterisks over the bars indicate a statistically significant difference between the early/late isolates in the specific pair(s) of CF strains, *P* < 0.05 in the paired Student *t*-test. The box below displays the statistical parameters obtained when analyzing the strains grouping them in the three collections (bacteremia, early CF isolates and late CF isolates). A Student *t*-test *P*-value < 0.05 was considered statistically significant for the differences among the collections mean values. NA, not applicable.

**FIGURE 3:**
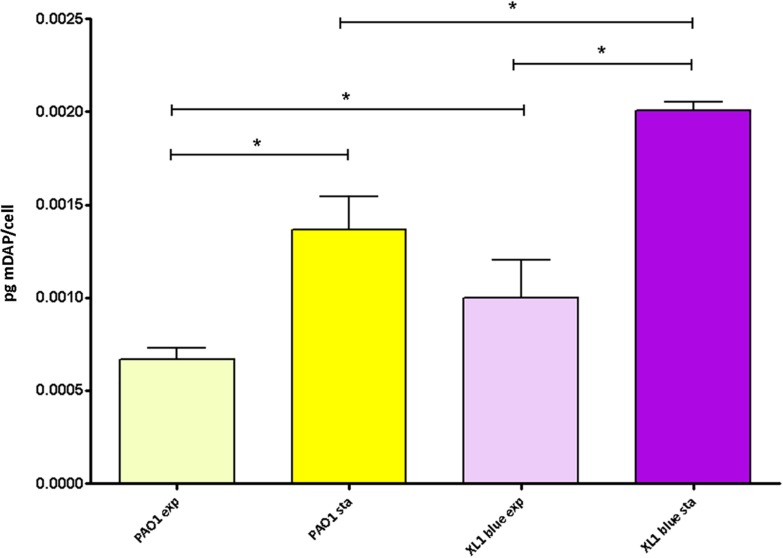
Peptidoglycan quantification through the titration of meso-diaminopimelic acid (mDAP) in the *P. aeruginosa* and *E. coli* reference strains at exponential vs. stationary growth phases. Each column represents the mean value of three independent determinations for each specific peptidoglycan, whereas the error bar represents the standard deviation (SD). ^∗^Students’ *t*-test *P*-value < 0.05.

Given the results displayed above, in which in spite of the likely high level of adaptation to each type/status of infection, the parameters showed a notable degree of uniformity among collections, we wanted to check if quantitatively speaking, these uniformity was also maintained. And as can be observed in the Figure 2, the amount of peptidoglycan per cell (quantified through the titration of mDAP) was very constant among all the strains as well. In fact, no statistically significant differences among the three collections were documented (*P* > 0.05). Regarding the values within the CF collection, although statistically significant differences between early/late isolates from the strains PAFQ06, PAFQ21-1088, and PAFQ24 were found, no significant trends along time when comparing the two collections (early-late CF) could be found (Figure 2). The only strain that stood out because of an odd value of mDAP/cell was PAFQ21-1088-03, with a value circa 3–4-fold below the mean value of the collections (Figure 2). Finally, as in the first section, we decided to check if *P. aeruginosa* could show an exaggerated degree of stability in the peptidoglycan when comparing different growth phases, this time from a quantitative perspective. As can be observed in the Figure 3, in which we also included the *Escherichia coli* XL1 blue strain for comparative purposes, both species displayed a significantly increased amount of peptidoglycan/cell in the stationary phase with regards their respective exponential phase (circa twofold in both cases, *P* < 0.05). Interestingly, the amount of peptidoglycan per cell of XL1 was significantly higher than that of PAO1 (circa 1.5–2 fold, *P* < 0.05) both in the exponential and stationary phases.

### Lack of Significant Differences in the Inflammatory Capacity of Peptidoglycans From Bacteremia vs. Cystic Fibrosis *P. aeruginosa* Strains

**FIGURE 4:**
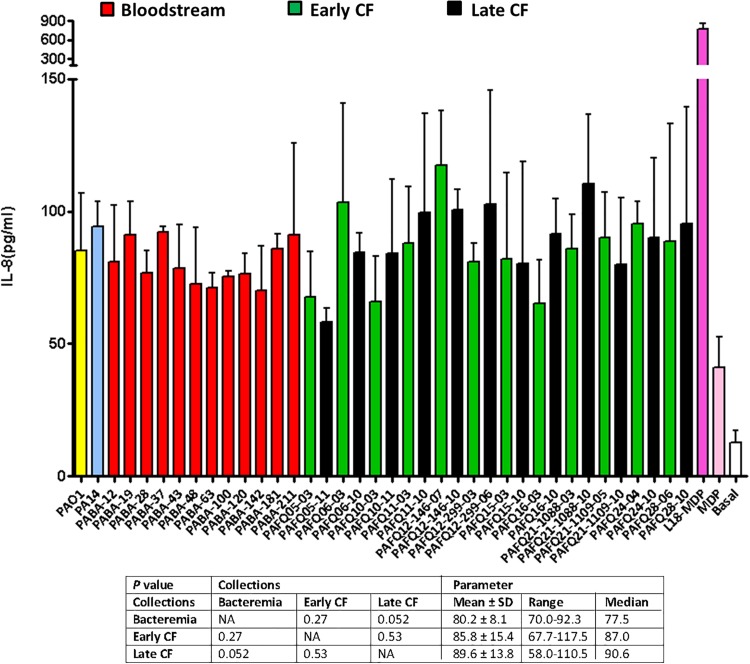
Pro-inflammatory capacity of the mutanolysin-digested peptidoglycans purified from the studied clinical *P. aeruginosa* strains. Each column represents the mean value of three independent determinations for each specific peptidoglycan, whereas the error bar represents the standard deviation (SD). In the CF pairs of isogenic isolates, the green columns correspond to early and the black to late isolates, respectively. All the bacteremia strains are displayed with the red columns. The wells with cell culture medium and no addition of peptidoglycan were used as basal controls, whereas those stimulated with 20 μg/mL of MDP or the synthetic derivative L-18 MDP were used as positive controls. The box below displays the statistical parameters obtained when analyzing the strains grouping them in the three collections (bacteremia, early CF isolates and late CF isolates). A Student *t*-test *P*-value < 0.05 was considered statistically significant for the differences among the collections mean values. NA, not applicable.

In a last approach to characterize the peptidoglycans from our collections, we determined their pro-inflammatory capacity, stimulating semi-confluent cultures of A549 cells with 20 μg/mL of each mutanolysin-digested purified peptidoglycan for 20 h, and quantifying the release of IL-8 as indicator. As can be observed in the Figure 4, the overall inflammatory capacity of all the tested strains was very similar; in fact, when performing the One-way ANOVA with *post hoc* Tukey’s multiple comparison test (obviously excluding the basal control and the MDP/L-18 or MDP-stimulated wells) a *P-*value > 0.05 was obtained, indicating that the whole set of peptidoglycans was homogeneous regarding the inflammatory power. Moreover, the values of IL-8 released by the cells stimulated with the peptidoglycans from reference strains PAO1 and PA14 were also very close to the mean values of the three collections (between 85 and 95 pg/mL of IL-8). For all the purified peptidoglycans, the *E*-toxate assay provided negative results, indicating that no contamination was present in the final samples, and that consequently, the measured inflammation exclusively corresponded to peptidoglycan detection-response. On the other hand, the LDH release determinations to quantify the cytotoxic capacity of the peptidoglycans during the 20 h incubations, revealed that the cell death was always below 5%, similar to the non-stimulated wells (data not shown), discarding a toxic effect of peptidoglycans *per se*.

**FIGURE 5:**
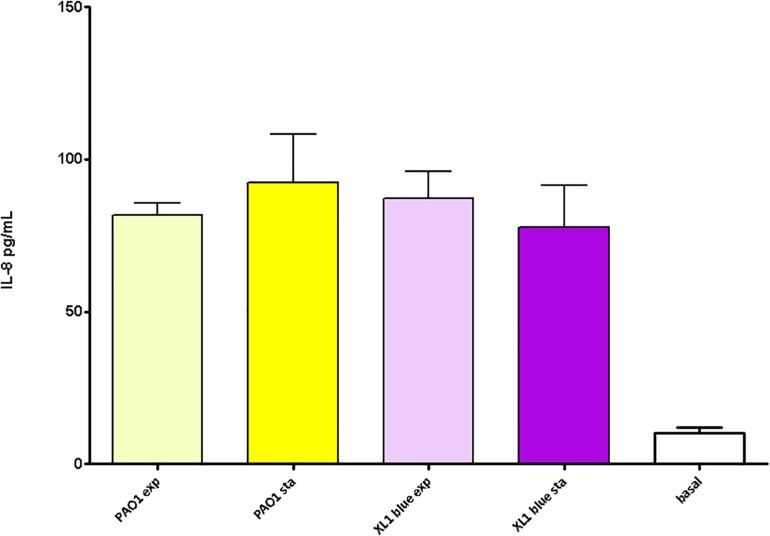
Pro-inflammatory capacity of the peptidoglycans purified from the *P. aeruginosa* and *E. coli* reference strains at exponential vs. stationary growth phases. Each column represents the mean value of three independent determinations for each specific peptidoglycan, whereas the error bar represents the standard deviation (SD). ^∗^Students’ *t*-test *P*-value < 0.05.

Finally, as for the previous sections, the peptidoglycans purified from stationary vs. exponential growth phases (both from PAO1 and XL1) were compared in terms of elicited inflammatory response. By this analysis we wanted to see if the uniform pro-inflammatory power displayed in Figure 4 was maintained when comparing different metabolic stadiums represented by the mentioned growth phases. Since some of the parameters obtained through HPLC were shown to be significantly different when comparing exponential vs stationary growth phases (e.g., muropeptides bound to OprI lipoprotein, muropeptides having a pentapeptide stem or average number of disaccharide units in the sugar chains, Table 4) it was plausible that the derived inflammatory capacity could also display some particularities. However, as can be observed in the Figure 5, the inflammation elicited by the PAO1 purified peptidoglycans obtained from stationary vs. exponential phases was very similar, a circumstance also observed for *E. coli* XL1 blue strain. Thus, no significant differences were observed among species and/or growth phases (*P* > 0.05).

## Discussion

Although the gram-negative peptidoglycan had a starring role during decades as object of study given its nature of essential structure, and later as target for β–lactams and basis for the regulation of resistance mechanisms such as the intrinsic β-lactamases (Greenwood, 1986; Juan et al., 2017; Dik et al., 2018), the studies analyzing its potential variations in large collections of strains are scarce (Boltaña et al., 2011; Wu et al., 2015). One could think that, given this indispensable role for survival counteracting the osmotic pressure, providing shape and allowing cells division, the strain-specific particularities should be exceptional because obviously, substantial changes in this conserved element could be often deleterious. Nevertheless, some studies have shown that in certain gram-negative species, the peptidoglycan can suffer punctual changes directly related with a better adaptation to the host in certain strains, and thus, the characterization of large collections of natural strains could be more attractive than initially suspected. In fact, the conception of the gram-negative peptidoglycan harboring a non-negligible degree of plasticity and variability is gaining weight in the last years, in terms of inter- and intra-species variability, but also referring to transient changes related with environmental stresses (Davis and Weiser, 2011; Cava and de Pedro, 2014; de Pedro and Cava, 2015). In this last regard, for instance, it has been shown that the CpxRA two-component system, responsible for sensing and controlling the cell envelope stress, could induce an strengthening of the cell wall thanks to an increase in the mDAP-mDAP cross-linkage (by upregulating the activity of certain L,D-transpeptidases), finally even enhancing the resistance to β-lactams in *E. coli* (Delhaye et al., 2016; Hugonnet et al., 2016). Additionally, certain mutation-driven modifications in peptidoglycans from specific strains of *Neisseria* spp., *Helicobacter pylori* and *Salmonella enterica* (Rosenthal et al., 1982; Swim et al., 1983; Dillard and Hackett, 2005; Wang et al., 2009, 2012; Davis and Weiser, 2011; Hernández et al., 2015; Juan et al., 2018), have been reported to cause increased resistance to lysozyme or bile, thus ameliorating the survival within the host. Similarly, some other changes in the peptidoglycan involving a diminished detection by the host’s receptors, thus favoring chronic persistence have been described also in *H. pylori* (Chaput et al., 2006; Wang et al., 2010). Finally, some peptidoglycans with very specific particularities do exist within gram-negatives, such as that of *Caulobacter crescentus*, with abnormally high levels of glycine in terminal position of stem peptides and no evidences of covalent links to Braun’s lipoprotein (Markiewicz et al., 1983).

Of course this kind of studies are much more abundant in gram-positives, in which the peptidoglycan, besides its greater thickness, is not protected by an outer membrane/lipopolysaccharides. This last circumstance enforces the need for selection of changes for resistance to lysozyme or avoid immune detection, to cite just two examples (Bishop et al., 2007; Davis and Weiser, 2011; Sukhithasri et al., 2013). But as stated before, the studies in this context with gram-negatives are not numerous, situation even more exaggerated in *P. aeruginosa*, which made highly pertinent the present work. Specifically in *P. aeruginosa*, previous works have proposed that its peptidoglycan can accumulate changes driving to a diminished inflammatory capacity, circumstance interpreted as a strategy to lower immune detection and ameliorate persistence in CF -evolved strains (Cigana et al., 2009, 2011). Nevertheless, our results suggest that at least for our collections, the peptidoglycans show a high degree of overall composition/structure stability among strains from different origins and with presumable different adaptations (acute vs. chronic, and early vs. late within CF collection). However, it must be stated that with our results we cannot infer if this uniformity is maintained *in vivo*, in which, for instance, the typical biofilm growth that CF strains display, or even the stress caused by the immune compounds could transitorily influence the peptidoglycan features. Future work will be needed to elucidate whether or not the growth in biofilm entails peptidoglycan changes in comparison with planktonic lifestyle, that would have gone unnoticed in the present study.

In any case, the parameters obtained through HPLC from our late CF strains revealed no evidences of changes like those described by Cigana et al. (2009, 2011): for instance, a reduction in certain types of dimers that were proposed to be due to alterations in the lytic transglycosylases of late strains. In fact, also in contrast with this latter work, none of our strains (neither from late CF nor from the rest of collections) showed a peptidoglycan with differential inflammatory capacity over cell culture. In this regard, one could argue that once purified, all the peptidoglycans should be equally inflammatory, since the cells were stimulated with the same amount of each mutanolysin-digested peptidoglycan (Schaub and Dillard, 2019a, b). But given that not all the peptidoglycan fragments show the same capacity to activate our specific receptors (NOD1 and NOD2) and cause the same level of inflammation (Girardin et al., 2003b; Kumar et al., 2013; Irazoki et al., 2019), a non-negligible possibility would be that certain strains showed alterations in the peptidoglycan hydrolases (Vollmer et al., 2008; van Heijenoort, 2011), which would affect the sacculus composition and the derived inflammatory capacity once the peptidoglycan was purified and digested with mutanolysin. In fact, some species of gram negatives (e.g., *Neisseria* spp. or *Bordetella pertussis*) are known to display particularities in the inflammatory capacity of their cell-wall fragments, mainly because of defects in their recycling machineries and/or singularities in the peptidoglycan-degrading enzymes (Rambow-Larsen and Weiss, 2002; Humann and Lenz, 2009; Chan and Dillard, 2016, 2017; Lenz et al., 2017; Irazoki et al., 2019; Schaub and Dillard, 2019a, b). In this same regard, Mallick et al. (2019) have recently shown that by inactivating different endopeptidases and D,D-carboxypeptidases in *E. coli*, the peptidoglycan inflammatory features suffer substantial alterations (Mallick et al., 2019). However, our results suggest that no significant alterations in the peptidoglycan-degrading pathways and thus, in the structure of the peptidoglycans, and consequently in the derived inflammatory power existed in our strains. Thus, the notable degree of inter-collection stability in the HPLC-derived parameters from the three collections suggested by our results would be also supported by their outstandingly uniform inflammatory capacity.

In spite of the mentioned absence of trends among collections peptidoglycans features’, it is true that some attention-calling particularities in certain isolates arose. For instance, the increased mDAP–mDAP crosslinking level in the strain PAFQ21-1088-03, circa fivefold higher that the three collections’ mean value. This characteristic has been related with increased levels of β–lactam resistance in *E. coli* (Desmarais et al., 2013; Hugonnet et al., 2016), but this seems to be not applicable to *P. aeruginosa*, since this strain was previously shown to be β–lactam susceptible (López-Causapé et al., 2013; Torrens et al., 2019, Supplementary Data Set S1). We tried to correlate the rest of attention-calling strain-specific traits highlighted in the results section with the phenotypic characterization we have recently published for the same strains (Torrens et al., 2019), which provides information regarding the susceptibility to different peptidoglycan-directed aggressions, such as the ceftazidime MICs, and the survival to lysozyme + colistin, and PGLYRPs + colistin treatments (Supplementary Data Set S1). However, the only statistically significant correlation appeared between the relative abundance of stem pentapeptides and the susceptibility to PGLYRP2 + colistin. More specifically, our results suggested an inverse correlation, i.e., an increase in the stem pentapeptides amount entails a decrease in the resistance against the action of PGLYRP2, once it reaches the peptidoglycan thanks to the permeabilizing action of subinhibitory colistin (Torrens et al., 2017, 2019). Interestingly, the same type of correlation was found with lysozyme + colistin treatment, although not overpassing the statistical significance threshold. These results suggest that the presence of stem pentapeptides favors the antipseudomonal power of the two mentioned immune proteins, both sharing a lytic activity (although in different sites of the peptidoglycan structure) (Torrens et al., 2017, 2019; Juan et al., 2018; Irazoki et al., 2019), on the contrary of PGLYRP1, PGLYRP3 and PGLYRP4, whose bactericidal power has been described to be independent of peptidoglycan binding and lysis (Kashyap et al., 2014, 2017). If this enhanced lytic power responds to an improved interaction of lysozyme/PGLYRP2 with the sacculus thanks to the longer stem peptides remains to be investigated in future. Another interesting idea that can be extracted from these results is that, consequently, *P. aeruginosa* would be more susceptible to the action of lysozyme or PGLYRP2 in the stationary phase, given the increase in the relative stem pentapetides amount compared to exponential phase that we have shown in our results. This would contrast with the well-known predominant activity of β–lactams during exponential growth phase (Tuomanen et al., 1986), which opens an interesting field to be investigated, in order to understand the mode of action particularities of the mentioned peptidoglycan-lytic proteins, with potential future therapeutic applications. However, similarly as stated above, what remains to be investigated is whether or not the differences we display regarding the increased activity of lysozyme/PGLYRP2 on the strains with higher abundance of stem pentapeptides, would be maintained if the treatments were applied to strains grown in biofilms.

Regarding the rest of particularities in the HPLC-derived parameters we found in punctual strains, although some of them could be related with previously described phenotypes, e.g., (i) an stiffer peptidoglycan when the relative abundance of dimers/trimers increases respect to monomers; (ii) a stronger outer membrane-peptidoglycan interaction when the proportion of muropeptides linked to Braun’s lipoprotein is high, (iii) a more elastic sacculus when the shorter are the sugar chains, etc. (Desmarais et al., 2013), since they were not following any trend among collections, and thus, no adaptive clues could be deduced, we did not investigate their potential molecular/genetic basis, which remain to be dissected in future. Also remains to be further investigated the odd amount of peptidoglycan/cell in the strain PAFQ21-1088-03, well below the rest of the strains.

As cited before, this overall uniformity in the peptidoglycans features from our clinical strains (also including the quantitative perspective), made us hypothesize that this structure could be, specifically in *P. aeruginosa*, outstandingly stable in comparison with other species, and thus, even display minor variations when comparing different metabolic stadiums. For this reason we decided to check if the changes in peptidoglycan structure reported to happen in *E. coli* during exponential vs. stationary growths could be softened in *P. aeruginosa*. In this regard, our results suggest that the dynamics of *P. aeruginosa* peptidoglycan modification were similar to those previously published for *E. coli* (Schwarz et al., 1969; Pisabarro et al., 1985) when comparing the two phases, but only for some of the parameters. Namely, increased levels of mDAP–mDAP bonds, muropeptides linked to OprI (Braun’s lipoprotein in *E. coli*) and crosslinking, and decreased sugar chain length, all referred to the stationary phase. On the contrary, an interesting particularity was the overall stability in the relative abundance of monomers, dimers and trimers between the two phases in *P. aeruginosa*, whereas for *E. coli* a significant decrease in monomers (obviously translated into an increase in dimers/trimers) was previously described for the stationary phase (Pisabarro et al., 1985), and confirmed by our assays. If these results respond to a more stable synthesis of PBPs during different growth phases in *P. aeruginosa* compared to *E. coli*, whose certain PBPs levels display notable differences among growth phases (Buchanan and Sowell, 1982; Pepper et al., 2006; Kong et al., 2010), remain to be further investigated. In any event, these results regarding growth phase could support those displayed above for the clinical strains (mainly regarding monomers, dimers and trimers), once more in the direction of a peptidoglycan structure more stable in *P. aeruginosa* than in other species. However, if this stability could be also applicable to comparisons between biofilm vs. planktonic growth remains to be elucidated in future. Following with these growth phase comparisons, which is also interesting from our results is the increased amount of peptidoglycan per cell in the stationary phase. This is perhaps logical if we think in an increased proportion of mature cells accumulating peptidoglycan bulk in order to facilitate the upcoming septation and cell division, but had not been experimentally investigated before, to our knowledge. Besides, our results regarding the amount of peptidoglycan per cell were in accordance with the previous works defending that *P. aeruginosa* cell-wall appears to be thinner than that of *E coli* (Matias et al., 2003; Turner et al., 2014). Finally as happened for the inflammatory capacity in the *P. aeruginosa* clinical strains, no significant differences appeared when comparing the peptidoglycans extracted in the exponential vs. stationary growth phases, suggesting that the changes accumulated in mature murein sacculus happen in peptidoglycan features that do not affect the NOD receptors activation levels (Girardin et al., 2003b; Irazoki et al., 2019). Interestingly, these same trends were observed for *E. coli* peptidoglycans as well.

This pioneer work reveals a perhaps unexpected high degree of stability in the peptidoglycan-related features in very diverse clinical strains of *P. aeruginosa*, including those highly adapted to the particular environment of the CF airway. In contrast with previous works defending that adaptation to CF could include the selection of changes driving to reduced inflammatory capacity of the peptidoglycan (Cigana et al., 2009, 2011), or several studies in other gram negative species showing that cell wall can accumulate adaptations to better resist within the host (Rosenthal et al., 1982; Swim et al., 1983; Costa et al., 1999; Dillard and Hackett, 2005; Chaput et al., 2006; Humann and Lenz, 2009; Wang et al., 2009, 2010, 2012; Davis and Weiser, 2011; Sukhithasri et al., 2013; Hernández et al., 2015; Frirdich et al., 2019; Schaub and Dillard, 2019a, b), our results with extensive collections of *P. aeruginosa* suggest that its peptidoglycan is conserved to a higher extent (even including comparisons between exponential and stationary growth phases), and probably, less susceptible of accumulating adaptive changes. Nevertheless, these results do not ensure that if analyzing the peptidoglycans of our strains grown in conditions mimicking their originative host features (i.e., presence of antibiotics, immune compounds, and mainly biofilm growth in CF), the same overall stability would have been observed. Thus, the potential transitory modifications that peptidoglycan may display during these mentioned conditions is an issue that remains to be investigated in a future work with this specific purpose. Moreover, we cannot discard that minimum and subtle particularities in the peptidoglycan composition of our strains could have gone unnoticed because of the detection limit of HPLC. But even with this circumstance, this is a very valuable first approach to the characterization of cell-walls from extensive *P. aeruginosa* clinical strains collections, a study never done before. On the other hand, we did find some attention-calling strain-specific intrinsic particularities, although their biological significance remains to be ascertained. Finally, the inverse correlation between the relative amount of stem pentapeptides within the murein sacculus and the resistance to immune lytic attacks against the peptidoglycan is also suggested by our results, making of this work a major step ahead to understand the biology of peptidoglycan from *P. aeruginosa* natural strains, hopefully useful in future for therapeutic alternatives design.

## Data Availability

All datasets generated for this study are included in the manuscript and/or the Supplementary Files.

## Author Contributions

AO and CJ designed and conceived the study. CJ, GT, ME-S, EP-P, and CC-M performed the experiments. CJ, GC, CL-C, and ER-M supervised the execution of the experiments. AO, CJ, GT, GC, CL-C, and ER-M analyzed and discussed the data. CJ wrote the manuscript with contribution from AO and review from AO, GT, GC, CL-C, and ER-M.

## Conflict of Interest Statement

The authors declare that the research was conducted in the absence of any commercial or financial relationships that could be construed as a potential conflict of interest.

## Supplementary Material

FIGURE S1 Representative chromatograms obtained in this study, and list of the muropeptides used for quantification.

DATA SET S1 Main features of the strains studied in this work. The strains are organized in two sheets: bacteremia (including also the *P. aeruginosa* reference strains PAO1 and PA14) vs. CF strains (displayed in pairs of early-late isolates). The Data Set shows all the relevant features of the strains, including molecular epidemiology, ceftazidime MICs and finally, the mean value ± SD from each strain regarding the bacterial survival after the following treatments: lysozyme + colistin (25 + 0.025 mg/L), PGLYRP1 + colistin (50 + 0.025 mg/L) and PGLYRP2 + colistin (50 + 0.025 mg/L) [data obtained from Cabot et al., 2011; López-Causapé et al., 2018; Torrens et al., 2019 (see References section)].
