# Supplementary figures and images for: Comparative Analysis of Peptidoglycans From Pseudomonas aeruginosa Isolates Recovered From Chronic and Acute Infections
Source: Front Microbiol. 2019 Aug 27;10:1868. doi: 10.3389/fmicb.2019.01868 (PMC6719521; doi:10.3389/fmicb.2019.01868)

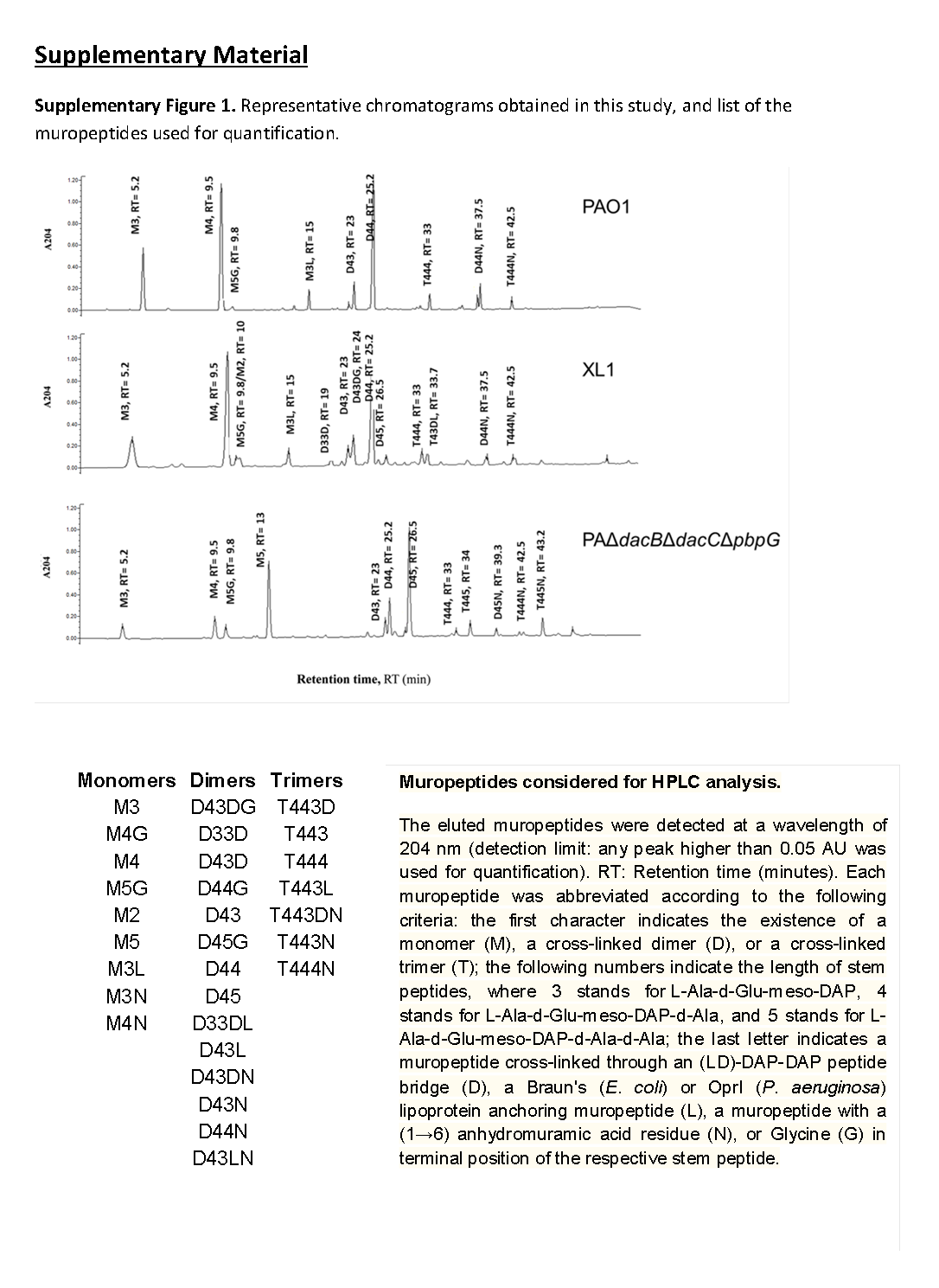

Supplement: FIGURE S1 — Representative chromatograms obtained in this study, and list of the muropeptides used for quantification. [file Image_1.TIF]
